# Gains and pains: a qualitative study on the implications of value-based health care for professionals

**DOI:** 10.1186/s12960-025-00972-x

**Published:** 2025-01-14

**Authors:** Veerle van Engen, Igna Bonfrer, Fabio Mieris, Malou Ensink, Anne Stiggelbout, Kees Ahaus, Martina Buljac-Samardzic

**Affiliations:** 1 Erasmus School of Health Policy & Management, Erasmus University Rotterdam, Rotterdam, The Netherlands; 2 Medical Decision Making, Department of Biomedical Data Sciences, Leiden University Medical Center, Leiden, The Netherlands

**Keywords:** Value-based health care, Healthcare professional, Motivation, Strain, Experience, Demands, Resources, Patient reported outcome measure, Netherlands, Qualitative

## Abstract

**Background:**

While aiming to optimize patient value, the shift towards Value-Based Health Care (VBHC) in hospitals worldwide has been argued to benefit healthcare professionals as well. However, robust evidence regarding VBHC’s workforce implications is lacking. This gap is problematic, as the motivation and health of healthcare professionals are central to the quality of care and crucial amidst contemporary workforce challenges. This study aims to qualitatively examine the implications of VBHC for healthcare professionals’ motivation, job strain, and ongoing participation in VBHC. In addition, it explores how these outcomes are regulated at both the individual and organizational levels.

**Methods:**

Semi-structured interviews were conducted with 26 healthcare professionals across six Dutch hospitals. Interviewees engaged in three VBHC activities: (1) value-based outpatient consultations and/or; (2) value-based quality improvement activities; as well as in; (3) VBHC implementation. Interview questions and data analysis were guided by the Job Demands–Resources model.

**Results:**

VBHC interacts with four themes perceived to affect professional’s motivation (perception of making a positive impact, enjoyability of job activities, personal development, and sense of community and support) and three themes perceived to affect job strain (workload, cognitive demands, and confidence). VBHC creates both gains (primarily increasing motivation; occasionally reducing strain) and pains (primarily increasing strain; sometimes reducing motivation). The perceived impact of VBHC depends on the fit between the individual, one’s activities in VBHC, the working conditions, and the pace of VBHC implementation. An observation that warrants attention is that healthcare professionals with a 'do-er' mentality and high ambitions to optimize patient value can become demotivated to continue advancing VBHC with the same intensity, particularly due to perceived slow progress.

**Conclusions:**

While VBHC is centered around patients, this study emphasizes that the needs, experiences and changing role identities of healthcare professionals cannot be overlooked in this transition. VBHC currently presents as a double-edged sword for healthcare professionals: resulting in both gains and pains. In the move to VBHC, it is crucial to maintain alignment between the individual, their job activities, the work environment, and the pace at which VBHC unfolds. This is essential for fostering and retaining motivated individuals, who are not only vital to the workforce but also pivotal in advancing VBHC.

**Supplementary Information:**

The online version contains supplementary material available at 10.1186/s12960-025-00972-x.

## Background

Health systems are moving to Value-Based Health Care (VBHC) to optimize ‘value’: patient-relevant outcomes relative to the resources used to achieve these over the full cycle of care [1, 2]. VBHC alters healthcare professionals' job activities [3, 4], often claimed positively [1, 5, 6]. However, robust evidence regarding VBHC’s workforce implications is lacking [3, 4, 7–9]. This gap is problematic as healthcare professionals play a pivotal role in VBHC [1, 2, 10], and the current workforce challenges require their retention [11]. Professionals’ motivation and health are linked to patient outcomes and employee retention [12–17]. Consequently, maintaining a motivated and healthy workforce in the move to VBHC is vital for healthcare systems worldwide [18].

VBHC is a multifaceted concept [2, 19], and hospitals have thus far implemented it in diverse and partial ways [8, 20–23]. Many hospitals focus on integrating value in patient discussions and pursuing value-based quality improvements [3, 8, 22–24]. Professionals often use data from Patient Reported Outcome Measures (PROMs) [25–27], which are structured surveys that enable patients to self-assess and report on their symptoms, functioning, and well-being [26].

While centered around optimizing patient value, it is also claimed that VBHC benefits the healthcare professional. The founders of VBHC, Porter and Teisberg, suggest that VBHC helps healthcare professionals to “*pursue the aims that led them to the profession in the first place.*” [1] (p.479). Teisberg later states: VBHC “*can be a powerful mechanism to counter clinician burnout*” [5] (p.683). Similar messages have been voiced by others: “*VBHC is about […] reducing the burden on professionals and improving satisfaction with their work*” [6] (p.4). However, these claims lack substantiation, and empirical studies indicate that healthcare professionals also encounter challenges in VBHC [3, 28–30]. Given the limited empirical focus on healthcare professionals in VBHC [4, 8], the implications for them remain poorly understood [3, 4, 7, 9].

This study aims to examine the perceived implications of moving towards VBHC for healthcare professionals. It focuses on exploring the mechanisms through which VBHC is perceived to affect professionals' motivation and job strain and seeks to understand how these factors affect their participation in VBHC. In addition, it explores how these outcomes are regulated at both the individual and organizational levels. These insights can help identify opportunities to better support professionals’ motivation and well-being in the value movement.

## Methods

### Theoretical model

**Fig. 1:**
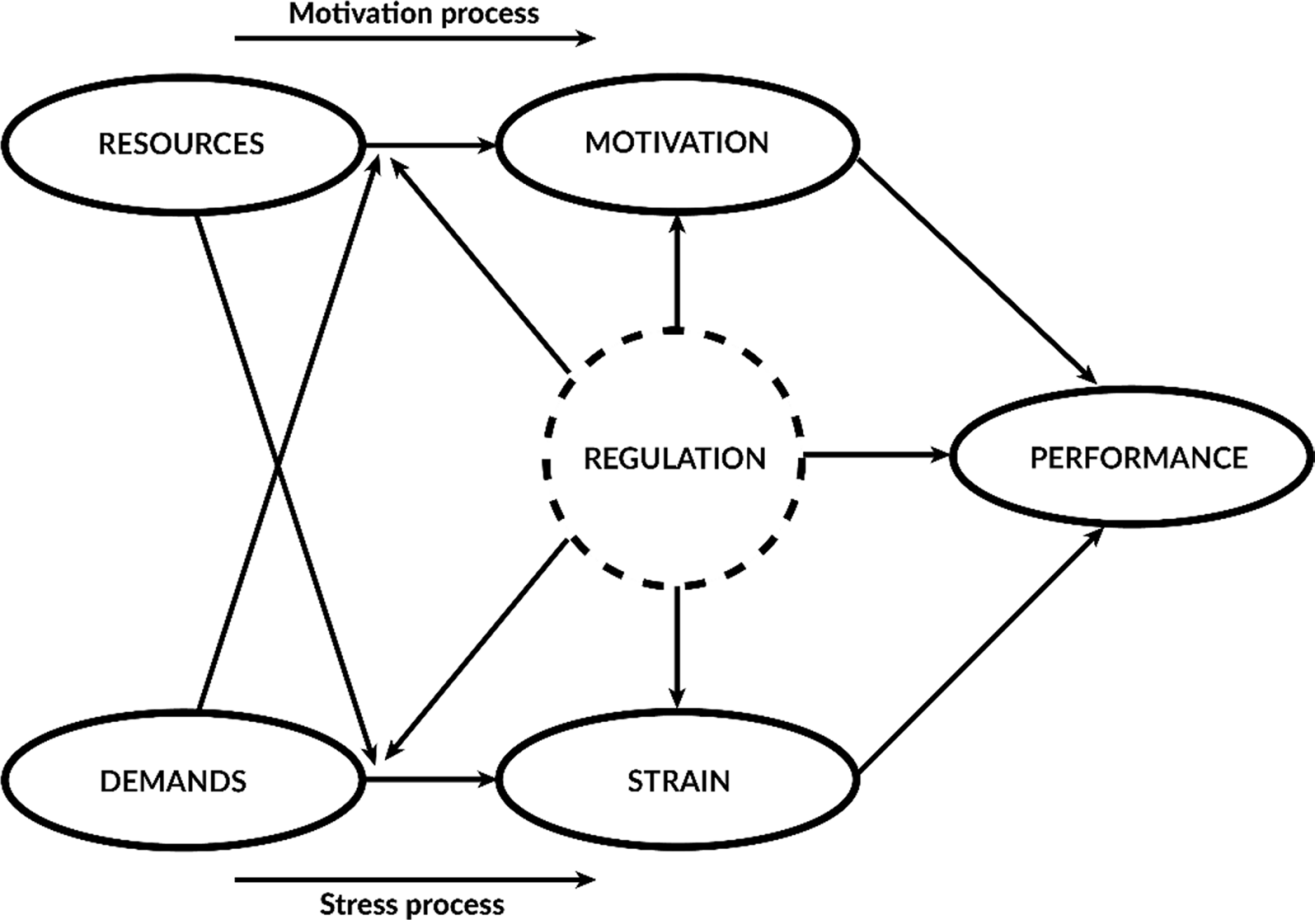
Adaptation from the extended JD–R model [32]

This qualitative study uses the Job–Demands–Resources (JD–R) model, which is widely employed in occupational health psychology [31–34]. The JD–R model is unique in its simultaneous focus on professionals’ motivation and strain [34, 35]. Motivation encourages professionals to engage in their work, while strain can hinder their ability to perform by depleting their energy and mental/emotional capacity. The JD–R model examines the mechanisms through which motivation and strain are influenced by demands and resources (Fig. 1). These can stem from the job, the individual, and the organization [32]. Resources can foster motivation and mitigate the impact of demands, while demands can increase strain and reduce the positive effects of resources. In addition, JD–R explores how motivation and strain affect professionals’ performance. Demerouti and Bakker (2023) expanded the JD–R model to include ‘regulation’ [32], which, in this study, refers to organizational and personal efforts aimed at enhancing motivation and mitigating strain for professionals.

We apply the JD–R model to explore the mechanisms through which VBHC is perceived to affect professionals' motivation and job strain, and the implications this has for their participation in VBHC [31]. In addition, using Demerouti and Bakker’s (2023) extended JD–R model [32], we study how individual and organizational regulation efforts influence these outcomes.

### Operationalizing VBHC

The JD–R model can serve both macro-level job analysis and micro-level examination of specific job activities [36]. In this study, we focus on three common VBHC activities implemented in Dutch hospitals and elsewhere [3, 8, 22–24]: *value-based outpatient consultations*, typically involving discussions with outpatients about their responses to PROMs to provide appropriate care; *value-based quality improvements*, primarily focusing on optimizing care processes based on outcome indicators, such as PROMs, often through benchmarking efforts with other healthcare centers; and *implementation efforts associated with both*, which aim to establish and sustain value-based outpatient consultations and value improvements within departments or for specific patient conditions [37], including establishing Information Technology (IT) and engaging colleagues.

### Data collection

Authors FM, ME, and VvE conducted semi-structured interviews with 26 Dutch healthcare professionals from 6 not-for-profit hospitals, including 2 university hospitals and 4 top-clinical hospitals. Two hospitals are part of the hospital group ‘Santeon.’ VBHC activities varied across the studied hospitals, with differences in their focus areas. Participants had at least 1 year of experience in value-based outpatient consultations or value-based quality improvements. In one hospital, a central VBHC coordinator facilitated the identification of potential interviewees, while in other cases, we relied on personal connections and snowball sampling. Purposive selection ensured representation from both nurses and physicians. Interview questions explored both positive and negative aspects of engaging in the selected VBHC-activities, their antecedents and consequences for the professionals themselves and their participation in VBHC. In addition, the questions explored how healthcare professionals and other stakeholders regulate these experiences to enhance professionals' motivation and well-being. Interviews were recorded and transcribed verbatim.

### Interviewee characteristics

Among the 26 interviewees, 46% were physicians, with the remainder being nurses. They were predominantly female (81%) and represented eight medical disciplines. All interviewees engaged in more than one of the three VBHC activities (20 in *value-based consultations,* 22 in *value-based quality improvements,* and all 26 in VBHC-*implementation*). Most could be considered pioneers and strong supporters of VBHC.

### Data analysis

Transcripts were deductively coded with Atlas.ti [38, 39]. A coding scheme was developed iteratively, with authors FM, ME, and VvE each coding eight interviews and discussing the results with the other authors. Following this, author VvE coded the entire data set using the developed coding scheme. This final coding process involved two steps, with multiple codes attached to quotes. First, text segments were coded based on their perceived impact on the JD–R outcomes motivation or strain, linked to a specific VBHC-activity and additional relevant codes (Table 1). In addition, transcripts were coded for basic information, including the hospital pseudonym, gender and function of the interviewee, and their role and level of experience in VBHC.

**Table 1:** Codes for initial coding

Coding step 1		
Codes: JD–R outcome category	Code: activity	Optional codes (if applicable)
**Motivation** (the extent to which professionals are willing and eager to engage in their work) • Increasing motivation • Decreasing motivation • Motivation unchanged **Strain** (the physical, mental, and emotional exhaustion that limits professionals' ability to engage in their work) • Increasing strain • Decreasing strain • Strain unchanged **Other** • Other outcome	**VBHC activities** • Value-based outpatient consultations • Value-based quality improvements • VBHC implementation • Other activity	**Performance** • Value-based consultations • Value-based quality improvement • VBHC implementation • Balance gains/pains **Interaction with** • Personal resources/characteristics • Organizational resources/characteristics • Ordinary job • Other **Regulation** • Personal regulation • Team regulation • Organizational regulation **Other** • Other (generic)

Second, data coded within motivation and strain were open and then axially coded [40] based on similarity to establish core themes. Resulting themes form the structure of the results and corresponding codes are presented in Table 2 in the respective section.

## Results

### Overview

We identify four themes that predominantly explain how VBHC is perceived to affect professionals' motivation (Sect. “Mechanisms affecting motivation”) and three themes that predominantly describe how it is perceived to impact their experienced strain (Sect. “Mechanisms affecting strain”). Table 2 presents these themes, outlines their mechanisms, and summarizes the associated codes. Additional file 1 contains exemplary quotes corresponding to each of the codes. The results conclude with a summary of implications for healthcare professionals’ performance in VBHC (Sect. “Performance: participation in VBHC”).

**Table 2:** Overview of results, illustrating the core code tree

Affected JD–R outcome	Theme	Mechanisms
Motivation: the extent to which professionals are willing and eager to engage in their work	Perception of making a positive impact	Increasing motivation • Genuinely supporting individual patients • Improving care for many patients collectively • Advancing VBHC
		Decreasing motivation • Constraints to making a positive impact • Doubting positive contribution of VBHC-efforts • Slow progress in achieving VBHC and optimizing value^a^
	Enjoyability of tasks	Increasing motivation • Increased richness and depth in consultations • Increased task diversity • Alignment of tasks with expertise and preferred challenge level
		Neutral • No changes in one’s tasks • Work remains equally enjoyable
		Decreasing motivation • Reduced time for valued tasks • Discontent with VBHC-related task attributes
	Personal development	Increasing motivation • Opportunities for personal development • Personal growth
		Decreasing motivation • Limitations in feedback
	Sense of community and support	Increasing motivation • Teamwork (internal) • Collaboration (external) • Organizational support
		Decreasing motivation • Unengaged colleagues^a^ • Limitations in organizational support^a^ • Systemic limitations within national healthcare system^a^
Strain: the physical, mental, and emotional exhaustion that limits professionals' ability to engage in their work	Workload	Increasing strain • Additional, uncompensated time investment
		Neutral • Unnoticeable changes in workload
		Decreasing strain • Efficient, streamlined processes
	Cognitive demands	Increasing strain • Data overwhelm and scatteredness
		Decreasing strain • Improved oversight (processual) • Ease from increased information availability
	Confidence	Increasing strain • Deviating from established standards • Perceived limitations in competence
		Neutral • Feedback on performance
		Decreasing strain • Confidence from increased information availability • Evidence of high-quality service delivery^b^

^a^These elements were associated with both decreased motivation and increased strain

^b^These elements were associated with both increased motivation and decreased strain

Overall, the results indicate that VBHC has a dual impact, creating both gains and pains. Gains primarily manifest in increased motivation and, occasionally, reduced strain. Conversely, pains are evident in increased strain and, at times, reduced motivation. Throughout the text, we discuss how professionals and organizations have sought to optimize gains and limit pains, indicated by the term ‘regulation.’

### Mechanisms affecting motivation

#### Theme 1: Perception of making a positive impact

All interviewees perceived that VBHC supports, or has the potential to support, a positive impact on patients' lives or healthcare overall, aligning with their professional goals. This gave them a sense of “*meaningfulness*” (interviewee 1), *“joy”* (interviewee 7) and *“fulfillment”* (interviewee 11). Those involved in value-based consultations felt that VBHC helped them genuinely support individual patients. For instance, interviewee 8 reflected: “[*Patients] don't want to hear ‘Your DAS score is 2.8; we need to change your medication.’ They want to talk about ‘I'm very tired,’ and then we address that.*” Verbal appreciation from patients and improved patient satisfaction scores reinforced professionals’ motivation.

Professionals involved in value-based quality improvement and VBHC implementation felt they were advancing healthcare and positively impacting many patients. Interviewee 23 stated: “*I feel that this approach enables me to have more organizational influence and ultimately make a greater impact, reaching more people than I would with seeing individual patients in the consultation room*”. They recognized their contributions through enthusiastic reactions from colleagues, acknowledgement as pacesetter in VBHC, and seeing materials or processes they developed adopted by other centers.

Conversely, interviewee 25 occasionally felt that her impact on patient-relevant outcomes was limited. She felt insufficiently able to address issues like fatigue or quality of life, which diminished her satisfaction with her work output. Moreover, observing low patient participation in PROMs led some professionals to question the value of PROMs for patients.

Nearly, all interviewees expressed frustration with the slow progress of VBHC implementation and the limitations in visible results. This was experienced as both demotivating and energy-draining. Some also perceived value-based quality improvements as unnecessarily slow, characterized by extensive discussion and preparation but limited action. Consequently, interviewee 12 expressed doubt: *“Sometimes you wonder if you are doing the right things because it feels like we are not getting anywhere.”* Interviewee 8 suggested that organizational regulation could be enhanced by highlighting progress: *“I think a lot is happening behind the scenes, but we don’t see it in the rheumatology clinic.”* Several interviewees described the slow pace as conflicting with their nature as ‘do-ers’ with high ambitions, leading to negative emotions. Interviewee 8 articulated feelings of: “*impatience and frustration, thinking, ‘Come on, let's move forward,' and also some disappointment, realizing that my expectations of achieving quick results were wrong.”* This frustration was compounded by perceived dependencies on others within the organization, which hindered their ability to expedite processes. To cope with the slow pace, interviewees regulated their expectations and emotions by accepting the situation and practicing patience. Moreover, interviewee 8, planned to participate *“very low-profile”* in the future value-based quality improvement activities to focus her scarce time on activities where she can make a greater impact, at times feeling inclined to *“drop out.”*

Furthermore, in value-based quality improvement, interviewee 23 had yet to experience any *“eye-openers,”* noting that cross-center differences in performance mostly stemmed from unequal registration rather than disparities in care quality. The lack of extramural use of PROMs and lack of attention to the cost component of VBHC were also seen as constraints to achieving impact.

#### Theme 2: Enjoyability of job activities

Nearly all interviewees enjoyed their activities in VBHC despite facing challenges and setbacks. Many noted an increased richness, both within and across their tasks. In value-based consultations, they appreciated the enhanced depth and comprehensiveness of conversations with patients: *“Talking about more than just their disease gives me more satisfaction”* (interviewee 11).

Several professionals valued increased diversity of tasks, expressing enthusiasm for pioneering, innovating, and enhancing care practices. Interviewee 23 explained: *“I wouldn't enjoy being confined to the consultation room alone. […]. [Seeking value-based quality] improvements has significantly contributed to my joy at work.”* Interviewee 17 added: *“I see something, I have an idea, and VBHC provides me with the opportunity to investigate it.”*

Some professionals appreciated how tasks were better aligned with their expertise and the desired level of challenge. Two physicians explained how a VBHC improvement cycle reorganized tasks to optimize the use of each person's expertise and time. This enabled them to focus exclusively on complex patients. While this increased cognitive demands, this reorganization was viewed positively due to extended consultation times, a unique practice in their department (organizational regulation). Resultantly, interviewee 4 noted:*“[it] makes consultations much more interesting, and you don't feel like you have to rush all the time.”* Furthermore, interviewee 9 appreciated the challenges associated with innovating: *“A little stress is okay; otherwise, it gets boring.”*

However, not all experiences with VBHC activities were uniformly positive. One interviewee described a neutral impact, as she already had high work satisfaction before VBHC. Three others noted limited change, as they felt they were already working in line with VBHC principles before the official implementation.

In addition to the mismatch between VBHC's pace of implementation and professionals' preferred pace, four interviewees reported a negative impact of VBHC on their joy in work. This often stemmed from how VBHC was organized locally. For example, a nurse described being tasked with sending PROMs to patients before their appointments, feeling that this responsibility detracted time from her ability to provide direct patient care. While cognitively understanding the relevance, emotionwise *“It really grabs me by the throat […] in that sense I do less of what I like to do”* (interviewee 13). Moreover, two interviewees expressed frustration with the increasing digitalization of their work due to PROMs and data-driven improvement activities. One of them regulated her motivation by intentionally avoiding using PROMs. In addition, during care pathway improvements, two interviewees regretted the ongoing discussions centered on financial implications and associated inter-departmental competition.

#### Theme 3: Personal development

Interviewees valued the increased opportunities for personal development that VBHC offered. They highlighted valuable feedback on team and individual performance, using aggregated outcome and experience data from their own patients. In one hospital, this was facilitated through a weekly ‘scorecard.’ This triggered curiosity and motivation: *“The most exciting email is the Monday morning scorecard mail […] Everyone is curious about it”* (interviewee 4), and “*that energy it provides, everyone wants to be the top performer and avoid being the lowest scorer*” (interviewee 14). However, motivation was tempered when feedback was infrequent or when outcomes were confusing and difficult to trace back to specific causes.

In addition, interviewees valued the personal growth they experienced through VBHC. Interviewee 12 described that VBHC’s focus on the whole person made her: “*a better, more complete doctor.*” Interviewees also reported developing skills in leadership, project management and change management, as well as gaining a deeper understanding of the healthcare system.

#### Theme 4: Sense of community and support

The collaborative nature of VBHC fostered increased teamwork and social support, both within individual hospitals and through inter-hospital collaborations. Interviewees appreciated *“enlarged networks*” (interviewee 6), “*closer connections*” (interviewee 21), “*more mutual understanding*” (interviewees 8), “*inspiration*” (interviewee 7), and the ability “*to rely on each other*” (interviewee 10), amongst others. Achieving desirable results together reinforced positive emotions. Some noted benefits from being part of a hospital group, which eased performance comparison and information exchange.

Beyond peer collaboration, several interviewees emphasized the importance of organizational support (regulation). Valued were training in VBHC activities, committed leadership, PROMs that were integrated into the Electronic Health Record (EHR), dedicated implementation time, and access to a data analyst.

However, challenges arose from unengaged colleagues, limited organizational support, and systemic constraints. These factors not only challenged professionals’ motivation but occasionally also increased their stress levels. Disengaged colleagues led to frustration and required significant energy to foster the necessary cooperation for VBHC. For instance, interviewee 8 expressed: *“Sometimes I felt like I was in a bubble, with none of my colleagues understanding what VBHC is.”* She described feelings of anger when the communication department inaccurately reported that *“WE do VBHC.”* Frequent staff turnover, both on the work floor and in management, intensified these challenges: “*You are constantly explaining and persuading new people*” (interviewee 16).

In addition, interviewees expressed lowered motivation and increased strain from feeling the need to validate VBHC without sufficient resources. Concerns were raised about the temporary nature of dedicated time for VBHC implementation, as activities like benchmarking will remain time-consuming. Nationally, barriers such as lack of leadership, IT and EHR limitations, and stagnant payment reform compounded these issues. As interviewee 12 put it: *“It feels like we are left in the cold; it doesn’t feel like we are doing it together as a nation.”* Another regretted the limited opportunities for innovation due to the financial risks associated with transitioning to VBHC being borne by their hospital.

### Mechanisms affecting strain

#### Theme 1: Workload

While interviewees noted that VBHC increased their overall workload, many found ways to manage it. In the consultation room, discussing PROMs and shared decision-making were seen as time-intensive activities. This posed challenges especially given high workloads and limited consultation times: “*You’re already busy, and then there’s more to do, which adds to the stress*” (interviewee 3). This challenge was exacerbated by delays in loading PROMs dashboards, the use of separate IT systems, and perceived redundant data entry.

One interviewee observed that potential workload reductions from VBHC, such as patients needing less care, were negated by persistent waiting lists, preventing professionals from experiencing a lighter workload. Interviewee 11 expressed frustration over the lack of focus on triaging patients using clinical and PROMs data: *“Currently, I still see all patients,”* highlighting this as an opportunity for organizational regulation*.* To regulate their workload, two interviewees chose not to discuss PROMs with patients, while three others only discussed them during calm shifts, making case-by-case decisions. However, one of them reflected that skipping PROMs lowered her satisfaction with care delivery, leading her to view this coping strategy as less than ideal.

Regarding value-based quality improvement, six interviewees reported working on these initiatives during evenings and free time. Identified areas of improvement led to additional work: “*That also causes some unrest. Doctors think, damn, I must arrange this too*” (interviewee 22). To regulate their workload, some interviewees increased their assertiveness and requested dedicated time: “*I stopped doing things in my own time*” (interviewees 19). Another interviewee coped by occasionally extending the workday, which helped her prevent taking work-related pressure home.

During VBHC implementation, key workload contributors included the challenging process of establishing IT, engaging colleagues, patients, and management, as well as conducting scientific research on VBHC. Moreover, success generated more work due to requests to help initiate VBHC for other patient conditions. To regulate their workload, interviewees involved colleagues and delegated tasks. Interviewee 25 coped with workload and change fatigue by becoming more selective in participating in VBHC initiatives: “*I’ll wait a bit and then judge: It’s nice, I participate. Or: It’s not nice, I refuse.”*

Conversely, five interviewees experienced time savings through VBHC. Within the consultation room, they noted that both patients and clinicians were better prepared, leading to more focused discussions: *“One can very specifically see and discuss what the patient wants to talk about instead of the standard routine”* (interviewee 8). In addition, optimized care pathways and protocols resulting from value-based quality improvement contributed to streamlined processes.

#### Theme 2: Cognitive demands

VBHC introduced additional cognitive demands for some healthcare professionals while alleviating these for others. During both value-based consultations and quality improvement activities, data overload contributed to increased cognitive strain. This overload arose from the multitude of patient and process indicators and a lack of oversight across different IT systems, leaving interviewee 25 feeling *“worn out”* at the end of his shift.

Conversely, interviewees also described how PROMs simplified their work processes, thereby reducing the need for mental effort. They found PROMs helpful in identifying priority areas in patient consultations, guiding discussions to cover all relevant topics, and facilitating conversations about sensitive issues. Two interviewees noted that aggregated PROMs data now assist them in educating patients and making decisions, creating a sense of ease and calm. Furthermore, value improvement activities were appreciated for making protocols and care pathways more transparent and clear.

#### Theme 3: Confidence

VBHC influenced emotional demands related to accountability in both negative and positive ways. Some interviewees felt insufficiently competent in using PROMs and analyzing data, which affected their confidence. Further, interviewee 21 noted that younger colleagues, trained under a philosophy emphasizing maximal standardization and risk reduction, experienced fear when delivering tailored care that deviates from established standards.

In contrast, interviewee 7 found relief in increased amount of data that VBHC provides. Besides PROMs data, an improvement activity in collaboration with the pharmacy allowed her to see whether patients have collected their medication, which gives her: *“confidence and ammunition for [patient] discussions.”* Three others appreciated VBHC’s benchmarking feedback, as it confirmed the quality of their care, providing reassurance and a sense of relaxation. Recognition as a best practice also motivated professionals to continue their work.

Some interviewees noted that their hospital effectively regulated a climate of psychosocial safety, making it not stressful to receive feedback on performance. This was established by allowing sufficient time before making data transparent externally, providing opportunities for improvement, and offering personal anonymity if desired.

### Performance: participation in VBHC

Most interviewees believed that the benefits of VBHC outweighed its demands, providing them with the strength and motivation to continue with VBHC. They expressed moderate optimism that future developments will improve the balance between gains and pains.

However, challenges to motivation and strain prompted some professionals to reduce their participation in VBHC. As discussed in the themes ‘enjoyability of work activities’ and ‘workload’, six interviewees reported not using PROMs or using them only occasionally in value-based consultations. This was primarily due to their preference for direct, tailored discussions with patients and the time constraints they faced.

Regarding VBHC implementation activities and care improvement efforts, three out of the 26 interviewees began to decrease their involvement. Key factors contributing to this decision included their high ambitions and desire for action, coupled with perceptions of slow progress, limited facilities and a lack of visible impact from their efforts, as highlighted in the themes ‘perception of making a positive impact’ and ‘workload.’

## Discussion

This study qualitatively examined how three Value-Based Health Care (VBHC) activities—value-based outpatient consultations, value-based quality improvement, and VBHC implementation efforts—are perceived to affect healthcare professionals’ motivation, job strain and ongoing participation in VBHC. In addition, it explored individual and organizational-level efforts to regulate professionals’ experiences, aiming to positively influence the implications of VBHC for them.

### Motivation and strain

We identified four themes that predominantly affect professional’s motivation: perception of making a positive impact, enjoyability of job activities, personal development, and sense of community and support. Within these themes, we observed mechanisms through which VBHC either increased, decreased, or left motivation unchanged. Similarly, professionals perceived three key themes—workload, cognitive demands, and confidence—to influence job strain.

It is challenging to make definitive claims about VBHC’s workforce implications, as these effects vary depending on the individual, the type of VBHC activity, local conditions, and the pace of implementation. Nevertheless, in broad strokes, all three VBHC activities currently appear to function as a double-edged sword, offering both gains (mainly increasing motivation, occasionally reducing strain) and pains (mainly increasing strain, occasionally reducing motivation).

### Regulation

Regulation efforts were identified at both the individual and organizational levels, aiming to positively influence professionals' perceptions of the implications of VBHC. Professionals primarily employed strategies aimed at finding workarounds for pains and enhancing their emotional and cognitive coping [41]. For instance, some professionals adjusted their expectations to be less affected by the slow pace of progress, and others stopped working on VBHC initiatives during personal time. However, addressing the root causes of pains, such as workload, was often seen as beyond their control. In addition, professionals crafted their job to enhance aspects of their work they found enjoyable. VBHC appears to diversify professionals’ tasks and create opportunities for personal development, allowing them to align their work activities with their strengths and interests. As professionals’ roles co-evolve alongside the ongoing development of VBHC and its supporting conditions, liminal space theory may provide a relevant perspective for helping professionals navigate this transitional period [53].

At the organizational level, we found examples of effective regulation aimed at enhancing job resources. These included providing well-functioning IT systems, access to data analysts, training, dedicated time for VBHC activities, and a safe climate. However, gaps in organizational support were also noted, extending to limitations in national leadership and data platforms.

### Performance: participation in VBHC

While professionals generally reported a positive balance of gains over pains, some described their participation in VBHC as suboptimal or intentionally reduced their involvement, highlighting the need for further attention. We found that professionals’ self-regulation strategies to cope with VBHC-related strain may sometimes conflict with the intended delivery of VBHC. For example, some professionals chose not to use PROMs to alleviate time pressures and limit the digitalization of their work.

Furthermore, while VBHC initially motivated and energized healthcare professionals with ambitions to work according to the principles of VBHC, maintaining these positive outcomes and avoiding disappointments appeared challenging. Three out of 26 interviewees in this study reported scaling back their efforts in implementing VBHC and value-based quality improvement. This reduction was primarily due to perceptions of slow progress and the belief that they could achieve greater and more immediate impact through alternative activities.

### Advancing professional’ motivation and wellbeing in VBHC

Although VBHC primarily centers on patients, our findings highlight the critical need to also consider the professional. Addressing their needs and experiences is essential to prevent disengagement from VBHC or negative responses to future innovations [42, 43]. It seems critical to optimize the fit [44, 45] between the individual, their job activities, the work environment, and the pace at which VBHC unfolds.

In terms of person-job fit, our findings indicate that VBHC supports certain values typically held by professionals, such as the desire to engage in meaningful work, which is a key driver of motivation [46]. We find evidence that PROMs data, both at the individual patient level and in aggregate, are valuable resources for making positive contributions to patients, as perceived by professionals [47]. However, we also identified instances where VBHC conflicted with personal values, as evidenced by some professionals’ aversion to the increasing digitization of their work. VBHC demands specific and often plural skills, including ongoing learning and collaboration with patients, as well as role identities that integrate patient-centeredness with resource stewardship. This necessitates professional development in terms of both skills and identity work [48–50]. In addition, since VBHC relies on healthcare professionals as 'drivers' of change [10], change motivation, leadership and change capabilities appear essential [50–52].

In terms of the environment, we found significant variation in the facilities and support available to professionals across different sites. This variation seems to reflect their differing perceptions of motivation and strain with regards to VBHC. Some interviewees reported feelings of isolation in their VBHC efforts, particularly when dealing with disengaged colleagues or facing limited organizational and national-level support, echoing findings from previous research [29, 30, 54]. This suggests a potential over-reliance on pioneering healthcare professionals to drive VBHC without adequate backing. Conversely, being surrounded by enthusiastic peers substantially contributed to motivation and energy, signaling an opportunity for organizations to focus on social dynamics and foster a collective commitment to VBHC [46]. Specific organizational resources valued by professionals are discussed in Sect. "Regulation". Moreover, dashboard tooling could be improved to satisfy professionals’ information needs while addressing issues related to data fragmentation and overload [55].

Finally, this study confirmed that slow VBHC implementation could pose challenges [54], especially for professionals with a 'do-er' mentality and high ambitions. Strategies such as highlighting achievements and behind-the-scenes efforts, and creating small, visible wins [56] can help manage this challenge. In addition, providing professionals with information on how complex changes like VBHC typically proceed can help set realistic expectations [57]. However, other literature noted that issues may also arise when implementation is perceived as rushed [4, 58], possibly due to differences in readiness and willingness among individuals. Empowering healthcare professionals to establish a suitable pace for themselves may not be a perfect solution, as ensuring alignment among team members is crucial to minimize friction; VBHC inherently requires collaboration.

### Limitations

The results of this study may be skewed due to the inclusion of predominantly VBHC enthusiasts among the interviewees. Enthusiasts are likely more receptive to VBHC’s ‘gains’ but may also experience greater ‘pains’ if VBHC fails to meet their hopes and expectations. Given the variation in VBHC implementation across local sites, the workforce implications may vary across a broader population. In several instances, value-based efforts focused solely on patient outcomes, neglecting resource considerations, which raises the question of whether these initiatives can truly be considered value-based. Factors related to implementation might diminish over time. Furthermore, physicians were overrepresented in this study compared to the typical ratio between employed physicians and nurses, which could have influenced the results. The predominance of females in our sample aligns with the higher proportion of women in the healthcare sector in the Netherlands [59]. Quantitative studies on the workforce implications of VBHC could usefully complement this qualitative work.

## Conclusion

Value-Based Health Care (VBHC) initiatives currently create both gains and pains for healthcare professionals. While VBHC is centered around patients, this study emphasizes that the needs, experiences, and evolving role identities of healthcare professionals also deserve attention within the value movement. It is crucial to optimize alignment between the individual, their job activities, work environment, and the pace at which VBHC unfolds. This is essential for fostering and retaining motivated individuals, who are not only vital to the workforce but also pivotal in advancing VBHC.

## Supplementary Information

Additional file 1.

## Data Availability

The interview data are available from the corresponding author upon reasonable request.

## Abbreviations

VBHC: Value-based health care
PROMs: Patient reported outcome measures
IT: Information technology
EHR: Electronic health record
