# Supplementary material for: Gains and pains: a qualitative study on the implications of value-based health care for professionals
Source: Hum Resour Health. 2025 Jan 14;23:3. doi: 10.1186/s12960-025-00972-x (PMC11734497; doi:10.1186/s12960-025-00972-x)
Supplement: Supplementary file 1 — Additional file 1. [file 12960_2025_972_MOESM1_ESM.docx]

## **Additional file 1**

Additional File 1 contains exemplar quotes for all codes, organized into two tables.

Table 1. Exemplar quotes for motivation/strain affecting codes

| **Affected JD-R outcome** | **Theme** | **Mechanisms** | **Codes** | **Exemplar quote** |
| --- | --- | --- | --- | --- |
| Motivation: the extent to which professionals are willing and eager to engage in their work | Perception of making a positive impact | Increasing motivation | Genuinely supporting individual patients | *“[by discussing the patient's responses to PROMs], the conversation took a surprising turn, which brought the patient a sense of relief. It gave me great satisfaction to have facilitated that for the patient”* (interviewee 7) |
|  |  |  | Improving care for many patients collectively | “*I feel that this approach enables me to have more organizational influence and ultimately make a greater impact, reaching more people than I would with seeing individual patients in the consultation room*”.  (interviewee 23) |
|  |  |  | Advancing VBHC | *“I feel motivated and enthusiastic when you see progress being made, such as having a PROMs dashboard that we can now use in practice”* (interviewee 6) |
|  |  | Decreasing motivation | Constraints to making a positive impact | **“***Then you dig into it, and it turns out to be due to some kind of duplicate entry or just a slight difference in how we record things. So it’s not like you’re discovering anything where you’d say, 'Wow, this is a real eye-opener. Now we can truly improve the value of care.' I don’t come across that very often, which makes me question whether we should continue investing so much time in this”* (interviewee 24) |
|  |  |  | Doubting positive contribution of VBHC-efforts | *“But in practice, I notice that you have to remind the patient about 20 times to fill out the questionnaire again. […]. I don’t get the sense that patients really feel the need for it, let me put it that way. […]. You’re doing something without being sure it’s really necessary, and that affects my motivation.”* (interviewee 15) |
|  |  |  | Slow progress in achieving VBHC and optimizing value * | Motivation-related quote:  *"We are, of course, very focused on delivering quick results and solutions, but this process just moves much more slowly. That can challenge my motivation at times and makes you feel a bit uneasy because it takes so long."* (Interviewee 8)  Strain-related quote:  **“***You have to wait for other steps in the process, and you have no influence over that. That’s actually the most frustrating part. It drains your energy and focus, constantly inquiring about the status, but there's nothing I can do about it*” (interviewee 12) |
|  | Enjoyability of tasks | Increasing motivation | Increased richness and depth in consultations | *“Talking about more than just their disease gives me more satisfaction”* (interviewee 11) |
|  |  |  | Increased task diversity | *I wouldn't enjoy being confined to the consultation room alone. […]. [Seeking value-based quality] improvements has significantly contributed to my joy at work.”* (interviewee 23) |
|  |  |  | Alignment of tasks with expertise and preferred challenge level | *“A little stress is okay; otherwise, it gets boring”* (interviewee 9) |
|  |  | Neutral | No changes in one’s task attributes | *“My work has remained relatively stable, as it largely depends on my fundamental attitude toward my job—at least, that’s how I view it. I always strive to maintain a patient-centered perspective, so VBHC has not changed that.”* (interviewee 7) |
|  |  |  | Work remains equally enjoyable | “*I already had a high level of job satisfaction, and with VBHC, I still feel the same. I'm not sure if there’s anything that could increase it further. I'm happy.”* (interviewee 10) |
|  |  | Decreasing motivation | Reduced time for valued tasks | *“I'm often busy managing questionnaires and other tasks, which leaves me with much less time for the patients themselves. […]. It really grabs me by the throat […] in that sense I do less of what I like to do”* (interviewee 13). |
|  |  |  | Discontent with VBHC-related task attributes | *“It constantly revolves around money, productivity, and FTEs, and there's not even a mention of quality, PROMs, or PREMs—zero focus on that. This makes me think, 'This isn't why I chose this profession.' If we could shift that focus, it would make my work two to three times more enjoyable: not having to deal with the constant tug-of-war between departments. Above all, we all want to implement VBHC and deliver the best care for our patients”* (interviewee 4) |
|  | Personal development | Increasing motivation | Opportunities for personal development | “*[Regarding receiving feedback on personal care delivery]. That energy it provides, everyone wants to be the top performer and avoid being the lowest scorer*” (interviewee 14). |
|  |  |  | Personal growth | *“I have developed myself immensely. I joined as a very young medical specialist, and when they said, 'This really seems like something for you,' I naively replied, 'I’m going to do it.' […] Project management and leadership—skills I hardly possessed at the beginning. This [growth] feels rewarding.”* (interviewee 19) |
|  |  | Decreasing motivation | Limitations in feedback | *“What I find challenging is when you can't fully trace where the data comes from; why the outcomes aren't as expected. That holds me back from engaging with the issue to improve it”* (interviewee 8) |
|  | Sene of community and support | Increasing motivation | Teamwork (internal) | *“What positive things has it brought me? A lot of wonderful people to collaborate with and the successes we've been able to celebrate together”* (interviewee 23) |
|  |  |  | Collaboration (external) | *“I think it motivates different parties to ask, 'Oh, how is someone else doing it? Can we learn from that? Are we doing things well ourselves?' I enjoy that, because ultimately, it can lead to new insights or ideas”* (interviewee 21) |
|  |  |  | Organizational support | “*Because you have a shared goal and means to row in the same direction, it gives you the strength of ten as an organization”* (interviewee 10) |
|  |  | Decreasing motivation | Unengaged colleagues * | Motivation-related quote:  *“Sometimes I felt like I was in a bubble, with none of my colleagues understanding what VBHC is” (interviewee 8)*  Strain-related quote:  *“I believe that getting the team on board is important, even the most important aspect, and it also requires the most energy to convince people that this is the right way to work*” (interviewee 5) |
|  |  |  | Limitations in organizational support * | Motivation-related quote:  “*I am disappointed in the support, but others are truly frustrated. They are no longer willing to engage in the change”* (interviewee 18)  Strain-related quote:  *“And because there isn’t a data analyst available, I spend my scarce free time analysing data in Excel. That’s not how things should be. A few times, all right, but not structural.”* (interviewee 16) |
|  |  |  | Systemic limitations within national healthcare system * | Motivation-related quote  “*The electronic health records do not support what is needed for VBHC, and the developers are by no means facilitating this. This has made me start the process with a heavy heart.”* (interviewee 1)  Strain-related quote  *“And what drains all my energy is the lack of strong national leadership regarding the digital support of healthcare. […]. Every hospital, and sometimes every department, is developing its own IT systems independently. […]. So much energy wasted. […].* *It feels like we are left in the cold; it doesn’t feel like we are doing it together as a nation” (interviewee 12)* |
| Strain: the physical, mental, and emotional exhaustion that limits professionals' ability to engage in their work | Workload | Increasing strain | Additional, uncompensated time investment | *“When you start, you don't realize how much work it entails, how much energy it takes, and what obstacles you'll encounter along the way”* (interviewee 11) |
|  |  | Neutral | Unnoticeable changes in workload | *“I haven't noticed much relief in my workload from that yet”* (interviewee 9) |
|  |  | Decreasing strain | Efficient, streamlined processes | *“One can very specifically discuss what the patient wants to talk about instead of the standard routine”* (interviewee 8) |
|  | Cognitive demands | Increasing strain | Data overwhelm and scatteredness | *“You can get a bit overwhelmed […] you have so many information sources to consider, and yes, it can be quite labour-intensive to gather all that information”* (interviewee 14) |
|  |  | Decreasing strain | Improved oversight (processual) | *“PROMs have really provided me with a sort of guideline for conducting my consultations, ensuring I cover all relevant from the patient’s perspective”* (interviewee 8) |
|  |  |  | Ease from increased information availability | *“I can now show the patient that X percent achieve these outcomes after the procedure, and this is what patients experience if they choose not to undergo it. This information makes it much easier for me to help patients with decision-making”* (interviewee 4) |
|  | Confidence | Increasing strain | Deviating from established standards | *“[younger colleagues are] raised with the philosophy to maximize standardization and to go to great lengths to minimize every small risk, then that [customization] can be really daunting*” (interviewee 22) |
|  |  |  | Perceived limitations in competence | *“I’m not quite sure how to use PROMs or how others are utilizing them, which sometimes makes me feel uncertain, and those thoughts weigh on me”* (interviewee 2) |
|  |  | Neutral | Feedback on performance | *“I haven’t really experienced stress from it. [Our hospital] has created a safe environment for us to do this. It shouldn’t be simply used as a tool for accountability”* (interviewee 12) |
|  |  | Decreasing strain | Confidence from increased information availability | *“I can now see whether patients have collected their medication. This gives* *me* *confidence and ammunition for [patient] discussions” (interviewee 7)* |
|  |  |  | Evidence of high-quality service delivery ** | *Motivation-related quote:*  *“We secretly find it really exciting to be considered a best practice, which is quite flattering. Hearing that we're doing so well motivates us to maintain that status. We don't want to be known as 'oh yes, they were once very good, but now others have taken their place.' No, this reassures us that we are doing the right thing and fuels our motivation to keep it up” (interviewee 9)*  Strain-related quote:  *“I feel much more relaxed now that I know my work is appreciated. That is reflected in the data when you're doing the right things.”* (interviewee 16) |

* These elements were associated with both decreased motivation and increased strain.

** These elements were associated with both increased motivation and decreased strain.

Table 2. Exemplar quotes for other codes

| **Theme** | **Code** | **Exemplar quote** |
| --- | --- | --- |
| Regulation | Personal regulation: individuals' self-management strategies to support their motivation and well-being | *“I try to keep my expectations and emotions in check regarding how things will go; otherwise, I end up disappointed every time."* (Interviewee 21) |
|  | Organizational regulation: policies and practices implemented at the organizational level to support employee motivation and well-being | *“At a certain point, I got access to a data analyst, and that made my work in VBHC so much easier and more enjoyable”* (interviewee 23 |
|  | Team regulation: policies and practices implemented at the team level to support employee motivation and well-being | *"We divide the tasks to ensure everyone is involved and the workload is evenly distributed."* (interviewee 20) |
| Performance | Value-based consultations | *"Sometimes I don't discuss the PROMs, and I end up regretting it afterward."* (interviewee 4) |
|  | Value-based quality improvement | *"Yeah, then I think, next time I'll just participate very low-profile."* (interviewee 8) |
|  | VBHC implementation | *"I've already indicated that if we are going to implement PROMs for the next condition, someone else will have to take the lead on that.”* (interviewee 15) |
|  | Balance gains/pains | *"I perceive the balance as positive and feel confident in maintaining it [VBHC-efforts]."* (interviewee 14) |
| Interaction with | Personal resources/characteristics | *"I am a do-er doctor, just like many others. We want to solve problems and see the results of our actions, which can conflict with the progression of VBHC”* (interviewee 26) |
|  | Organizational resources/characteristics | *"Our PROMs dashboard is not yet integrated into the electronic health record (EHR), so we have to open a separate dashboard, which makes everything much more cumbersome."* (interviewee 24) |
|  | Ordinary job | *"This all adds to our already very demanding jobs."* (interviewee 1) |
| Other | Other activity | "I am now also involved with home monitoring." (interviewee 5) |
|  | Other outcome | *"I am curious whether VBHC encourages more people to pursue careers in healthcare."* (interviewee 19) |
|  | Other (generic) | *"There are still so many questions surrounding VBHC. Is what we are doing now truly VBHC? If not, then what is? What does the future look like? What does this mean for our work?"* (interviewee 17) |
